# Lnc-AIFM2-1 promotes HBV immune escape by acting as a ceRNA for miR-330-3p to regulate CD244 expression

**DOI:** 10.3389/fimmu.2023.1121795

**Published:** 2023-02-09

**Authors:** Chengxia Xie, Shengjie Wang, He Zhang, Yalan Zhu, Pengjun Jiang, Shiya Shi, Yanjun Si, Jie Chen

**Affiliations:** Department of Laboratory Medicine, West China Hospital, Sichuan University, Chengdu, China

**Keywords:** lncRNA, ceRNA, chronic hepatitis B, immune escape, CD244

## Abstract

Chronic hepatitis B (CHB) virus infection is a major risk factor for cirrhosis and hepatocellular carcinoma (HCC). Hepatitis B virus (HBV) immune escape is regulated by the exhaustion of virus-specific CD8^+^ T cells, which is associated with abnormal expression of negative regulatory molecule CD244. However, the underlying mechanisms are unclear. To investigate the important roles of non-coding RNAs play in CD244 regulating HBV immune escape, we performed microarray analysis to determine the differential expression profiles of long non-coding RNAs (lncRNAs), microRNAs (miRNAs), and mRNAs in patients with CHB and patients with spontaneous clearance of HBV. Competing endogenous RNA (ceRNA) was analyzed by bioinformatics methods and confirmed by the dual-luciferase reporter assay. Furthermore, gene silencing and overexpression experiments were used to further identify the roles of lncRNA and miRNA in HBV immune escape through CD244 regulation. The results showed that the expression of CD244 on the surface of CD8^+^ T cells was significantly increased in CHB patients and in the co-culture system of T cells and HBV-infected HepAD38 cells, which was accompanied by the reduction of miR-330-3p and the elevation of lnc-AIFM2-1. The down-regulated miR-330-3p induced the apoptosis of T cells by lifting the inhibition of CD244, which was reversed by miR-330-3p mimic or CD244-siRNA. Lnc-AIFM2-1 promotes the accumulation of CD244, which is mediated by decreased miR-330-3p, and then reduced the clearance ability of CD8^+^ T cells to HBV through regulated CD244 expression. And the injury in the ability of CD8^+^ T cells to clear HBV can be reversed by lnc-AIFM2-1-siRNA, miR-330-3p mimic, or CD244-siRNA. Collectively, our findings indicate that lnc-AIFM2-1 on CD244 by acting as a ceRNA of miR-330-3p contributes to HBV immune escape, which may provide novel insights into the roles of interaction networks among lncRNA, miRNA, and mRNA in HBV immune escape, highlighting potential applications of lnc-AIFM2-1 and CD244 for diagnosis and treatment in CHB.

## 1 Introduction

Chronic hepatitis B (CHB) infection continues to be a major health burden globally. Two billion people worldwide had contact with hepatitis B virus (HBV), with more than 290 million chronic HBV infections (1, 2). HBV is a noncytopathic virus, a double-stranded DNA virus, which needs to escape from the hosts’ immune surveillance to survive (3, 4). Immune escape of the virus is not only related to its gene mutation, but also the host immune of T cell response to the virus (5). As the potent immune system clears the virus, the liver mainly presents as acute and self-limiting hepatitis (6). In contrast, the virus escapes the host immune response with CD8^+^ T cell exhaustion, causing chronic hepatitis, and even progressing to cirrhosis and hepatocellular carcinoma (7). Therefore, repressing the occurrence of CHB has become a major breakthrough in treating hepatitis B and reducing the morbidity and mortality of cirrhosis and primary hepatocellular carcinoma. However, the mechanism by which antiviral CD8^+^ T cells exhaustion plays this role in HBV immune escape is unclear.

The overexpression of signaling lymphocyte activation molecule family member 4 (SLAMF4, CD244), which is a transmembrane protein present on immune cells, enhances CD8^+^ T cells depletion in CHB (8, 9). The ligand of CD244 is CD48, which is expressed broadly on hematopoietic cells (10). Under the stimulation of antigen-specific signals delivered through the T cell receptor (TCR), and CD244, as a co-stimulatory signal molecule, transmits the second signal and mediates the regulation of immune tolerance after being cross-linked with ligand CD48 (11). The interaction between programmed cell death receptor 1 (PD-1) and its ligand PD-L1 has been proved to play an important role in inducing hepatitis C virus (HCV) (12, 13) and HBV (14) infected T cell failure and apoptosis. Although high co-express of CD244 and PD-1 on CD8^+^ T cells, blocking CD244 or CD48 pathway could restore normal immune function of T cells independently of PD-1 (8). Hence, CD244 may be another potential target of immunotherapy for chronic viral infection. Further exploration of the molecular regulation mechanism of CD244 in mediating the depletion of effector T cell function and viral immune escape is likely to be the focus of effective control of HBV persistence and malignant progression.

More recent studies have shown that non-coding RNAs (ncRNA), including microRNA (miRNA) and long non-coding RNA (lncRNA) may also play a significant regulatory role in HBV infection (15–17). MiRNAs participate in many vital biological processes, such as cell signal transduction and immune response, through regulating target mRNA expression (18). Long non-coding RNA (lncRNA) is another non-coding RNA molecule containing more than 200 nucleotides (19). Interactions between lncRNAs and miRNAs are predicted because lncRNAs can act as sponges or inhibitors of interacting miRNAs (20). As a class of endogenous competitive RNAs (ceRNAs), lncRNAs can mediate gene expression by acting as miRNA sponges (21). According to previous reports, infection of viruses, such as tuberculosis, induces CD8^+^ T cells to upregulate CD244 and lncRNAs of the CD244 signaling pathway epigenetically regulate CD8^+^ T cell immune responses (22).

Thus, we hypothesize that the evidence of interaction between ncRNA and CD244 can help elucidate functional relationship between intracellular and intercellular molecules, thereby providing insights into biological processes, pathways and interaction networks that are critical to HBV immune escape. The present study was undertaken to specifically address how CD224 of T cells is involved in the process of HBV immune escape.

## 2 Materials and methods

### 2.1 Study subjects

The present study enrolled 20 CHB patients and 23 patients with spontaneous clearance of HBV (SC HBV) at West China Hospital of Sichuan University from March 2020 to September 2020. Chinese Medical Association guideline for the diagnosis of CHB: positive for HBsAg and/or HBV-DNA more than 6 month; spontaneous patients were enrolled follow: negative for HBsAg, positive for anti-HBs and anti-HBc, alanine aminotransferase (ALT) < 50 IU/L and aspartate aminotransferase (AST) < 45 IU/L. The study was approved by the Research Ethics Committee of West China Hospital of Sichuan University. Informed consent was obtained from all patients enrolled.

### 2.2 RNA extraction and microarray assay

The peripheral blood mononuclear cells (PBMCs) were obtained from three patients with SC HBV and three patients with CHB above mentioned. Total RNA was isolated using miRNeasy Mini Kit (QIAGEN, Hilden, Germany) according to the manufacturer’s instructions.

RNA quality assessment was performed using agilent 4200 platform. Detection of lncRNA, RNA and miRNA were performed by Gminix Informatics (Shanghai, China). Affymetrix Human Transcriptome Array 2.0 was used for differentially expressed lncRNAs and mRNAs detection, while Affymetrix miRNA 4.0 was used for miRNAs detection. The raw data for the microarray was uploaded to Gminix-Cloud Biotechnology Information (GCBI; http://www.gcbi.com.cn/gclib/html/index) for further study, and then analyzed the data using Robust Multichip Analysis algorithm. Threshold used to screen differentially expressed lncRNAs, mRNAs and miRNAs were fold change > 1.2 with a *P*-value < 0.05.

### 2.3 GO and KEGG pathway analysis

The Gene Ontology (GO; www.geneontology.org) enrichment was calculated to assess the biological process, cellular component and molecular function of the differential expression genes found (23). The differentially expressed mRNAs were mapped to terms in the GO database, and the number of genes of each term was calculated. The *P* < 0.05 denoted the significance of GO term enrichment in the deregulated expressed genes. Pathway analysis was used to investigate the differentially expressed mRNAs according to the Kyoto Encyclopedia of Genes and Genomes (KEGG; http://www.genome.jp/kegg/) database (24). Fisher’s exact test and χ^2^ test were used to select significant GO categories and KEGG pathways, and the threshold of significance was defined by *P* < 0.05 (the FDR was used to correct the *P* value).

### 2.4 Reverse transcription and qRT-PCR

Total RNA was extracted from blood or cells, and then dissolved in Trizol reagent (Invitrogen, USA) according to the kit’s instruction. The cDNA was synthesized by reverse transcribing 1 μg RNA using a Prime Script RT regent kit (TaKaRa, Japan). For the RT-qPCR, the primer sequences were designed and synthesized by Invitrogen. The amount of cDNA was amplified using a SYBR Premix Ex Taq II (TaKaRa) with primers for CD244, miR-330-3p and lnc-AIFM-2-1. GAPDH served as a loading control.

### 2.5 Flow cytometry

Cells were detached from the blood samples of EDTA anticoagulation or the cell culture dish with accutase (GE Healthcare), washed once with phosphate-buffered saline (PBS) and fixed in 70% ice-cold EtOH. The cell surface markers, including CD45, CD3, CD4, CD8, CD16 and CD244, were analyzed by three- or four-color flow cytometry, using fluorochrome-conjugated monoclonal antibodies (PerCP/FITC/APC/PE/BV510/BV421 anti-Human, BD). Apoptosis of T cells was examined by staining with Annexin V-FITC/PI (Beyotime Biotechnology, China). Fluorescence intensity was measured with FACSCanto Flow Cytometer and analyzed with FACSDiva Software (BD FACSCalibur, USA) and FlowJo (TreeStar Inc.) or Flowing Software (Turku Centre for Biotechnology).

### 2.6 Cell culture and transfection

Jurkat, HepAD38 and LO2 obtained from American Type Culture Collection (ATCC) were cultured in Dulbecco’s modified Eagle’s medium (DMEM) supplemented with 10% fetal bovine serum (FBS) and 1% penicillin-streptomycin under 37°C with 5% CO_2_ conditions. In order to study the effect of HBV on hepatocytes mediated by T cells, Jurkat cells were co-cultured with HepAD38, a hepatocyte line infected with HBV, or LO2 cells, a normal hepatocyte line. And then the apoptosis of cells, clearance of HBV and relative gene expression were gradually detected after 24 h. In order to investigate the effect of microRNA-330-3p on the clearance of HBV mediated by T cells, antisense oligonucleotides (ASO) for miR-330-3p or miR-330-3p mimics was transfected into a co-cultured system of Jurkat and HepAD38 using Lipofectamine 2000 (Invitrogen, 11668500) according to the manufacturer’s instructions. For the knockdown of CD244 or lnc-AIFM2-1, Jurkat and HepAD38 cells were performed siRNA oligonucleotide of CD244 or lnc-AIFM2-1. For the overexpression of CD244 or lnc-AIFM2-1, the plasmid of pcDNA-3.1-CD244 or pcDNA-3.1-lnc-AIFM2-1 was transfected into the co-cultured system of Jurkat and HepAD38.

### 2.7 Luciferase reporter assay

HEK293T cells were cultured in a 48-well cell culture dish, reaching a density of 70% confluence by the time of first transfection. ASO-miR-330-3p or miR-330-3p mimics was transfected by Lipofectamine 2000 (Invitrogen, 11668500). The medium was changed to DMEM supplement with 10% FBS after 6 h. 12 h after the first transfection, a pmirGLO plasmid (Promega) containing WT/Mut sequence of CD244 or lnc-AIFM2-1 was transfected by Lipofectamine 2000 reagent. Six hours after transfection, the medium was changed to DMEM supplement with 10% FBS, and the cells were cultured for 48 h. To test the luciferase activities, the HEK293T cells were collected and detected by a Dual-Luciferase reporter assay kit (Biyotime, RG009).

### 2.8 Western blotting analysis

The protein level of CD244 was determined by Western blotting. HepAD38 cells and Jurkat cells were lysed with RIPA lysis buffer for 30 min on ice, followed by differential centrifugal for fractionation. The protein concentrations were determined by a BCA protein assay kit (Thermo). Equal amounts of 20 μg proteins were separated by SDS-PAGE and transferred to a polyvinylidene fluoride (PVDF) membrane (Bio-rad). PVDF membranes were blocked in 5% nonfat milk for 1 h at 37°C, then incubated with primary antibody (BV421 mouse anti-Human CD244, 1:1000, BD) for overnight at 4°C and appropriate secondary antibody for 1 h at 37°C. The blot was visualized using a Western Lightning™ chemiluminescence reagent (PerkinElmer, USA) and analyzed by IPP 7.0 software.

### 2.9 ELISA for HBV detection

The expression levels of HBV-DNA, HBsAg and IFN-γ were detected by ELISA kit (Createch Biology, Tianjin, China). 50 μl samples and control samples were added into separate wells. The wells were incubated with Ab-HRP conjugates for 1 hour at 37°C, washed 5 times with PBST. 100 μL of substrate solution was added to each well and the reaction was quenched after 15 min incubation in darkness. Absorbance at 450 nm was measured using a microplate reader (BIO-RAD, USA).

### 2.10 ceRNA analysis

According to the ceRNA hypothesis, lncRNAs compete for the same miRNA response elements and act as ‘molecular sponges’ for miRNAs, thereby regulating the derepression of all target genes of the respective miRNA family. The miRNA targets on mRNA 3′ untranslated regions (UTR) and lncRNA were calculated using the PITA algorithm (http://genie.weizmann.ac.il/pubs/mir07).

### 2.11 Statistical analysis

All data were analyzed using GraphPad Prism version 7 (https://graphpad.com) and shown as mean ± standard error of mean. The Student t test was performed to analyze the microarray and qRT-PCR data. ANOVA was used to compare continuous variables. The comparisons between groups were made using two-way analysis with Turkey’s multiple comparisons test. The value *P* < 0.05 was considered as statistically significant.

## 3 Results

### 3.1 Identification of differentially expressed mRNAs, miRNAs and lncRNAs in CHB and spontaneous clearance of HBV

There were 20 patients with CHB and 23 patients with SC HBV in our study. As for the liver function parameters, the average HBsAg and HBeAg levels of CHB group were obviously higher than SC HBV group (**Supplementary Table 1**).

**Figure 1:**
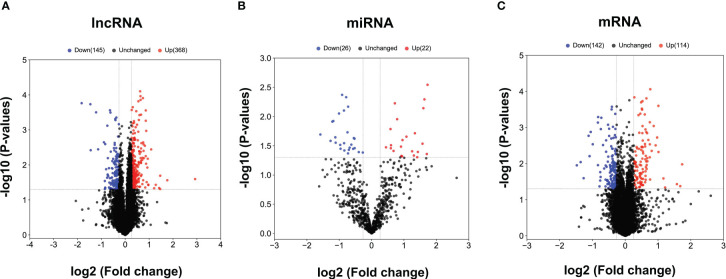
The volcano plots of differentially expressed lncRNAs, miRNAs and mRNAs between CHB patients and SC HBV controls. **(A)** Results of differentially expressed lncRNAs expression analysis between CHB (n = 3) and SC HBV (n = 3) patients. **(B)** Results of differentially expressed miRNA expression analysis between CHB (n = 3) and SC HBV (n = 3) patients. **(C)** Differentially expressed miRNA expression analysis between CHB (n = 3) and SC HBV (n = 3) patients. The abscissa is log2 (FC value) and the ordinate is -log10 (*P* value). Blue dots are downregulated genes, red dots are upregulated genes, and black dots are genes that were the same between the two groups.

The obtained RNAs expression profiles were analyzed by microarray analysis. A total of 513 lncRNAs, 256 mRNAs and 48 miRNAs were found to be differentially expressed (DE) in patients with CHB compared with patients with SC HBV (fold change > 1.2 and *P* < 0.05). Among them, 368 DE lncRNAs, 114 mRNAs and 22 miRNAs were upregulated, while 145 lncRNAs, 142 mRNAs and 26 miRNAs were downregulated in CHB patients compared with SC HBV patients. The volcano plots of these RNAs indicated that the DE RNAs can distinguish between CHB patients and SC HBV patients (**Figures 1A–C**).

### 3.2 HBV infection induced CD244 signaling expression on CD8^+^ T Cells in CHB patients

**Figure 2:**
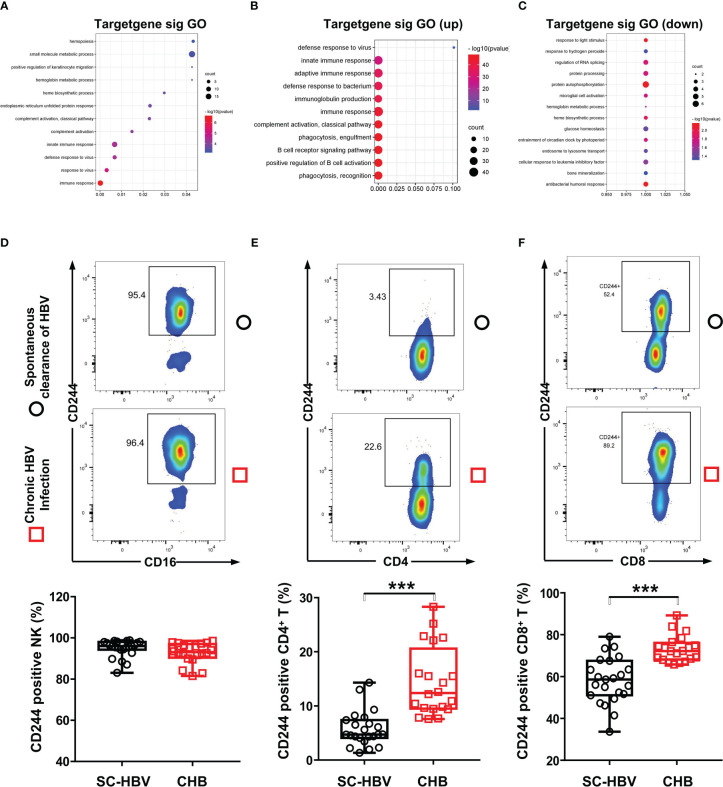
CD244 signaling expression on immune cells in CHB patients and SC HBV controls. **(A)** GO enrichment analysis of all significant genes, (*P* < 0.05). GO: Gene Ontology. **(B)** GO analysis for upregulated genes classified as signaling molecules. **(C)** GO analysis for downregulated genes classified as signaling molecules. **(D-F)** Activation and expansion of CD244 on NK cells **(D)**, CD4^+^ T cells **(E)** and CD8^+^ T cells **(F)** were analyzed by flow cytometry. Data (CHB, n = 20; SC HBV, n = 23) were analyzed using Student t test, *** *P* < 0.001.

Furthermore, the DE mRNAs and miRNAs were analyzed in heat map (**Supplementary Figure 1**). Then, we investigated the biological functions of DE RNAs *via* GO analysis, which supported the role of immune responses pathways evidenced in the GO term analysis including T cell receptor signaling pathway (**Figures 2A–C**). To determine whether CD244 signaling is involved anti-HBV immune responses, we examined CD244 expression levels in CD4^+^ T cells, CD8^+^ T cells, and NK cells. There is experimental strategy and cytometry plots (**Supplementary Figure 2**). Flow cytometric analysis showed that, compared with SC HBV patients, HBV infection induced significant increases in CD244^+^CD8^+^ T cells and CD244^+^CD4^+^ T cells, but not CD244^+^CD16^+^ NK cells (**Figure 2D**). In addition, percentages of CD244^+^CD8^+^ T cells were much higher than those of CD244^+^CD4^+^ T cells in PBMCs from either SC HBV patients or patients with CHB (**Figures 2E, F**).

### 3.3 HBV infection induced CD244 overexpression promoted T cells apoptosis

**Figure 3:**
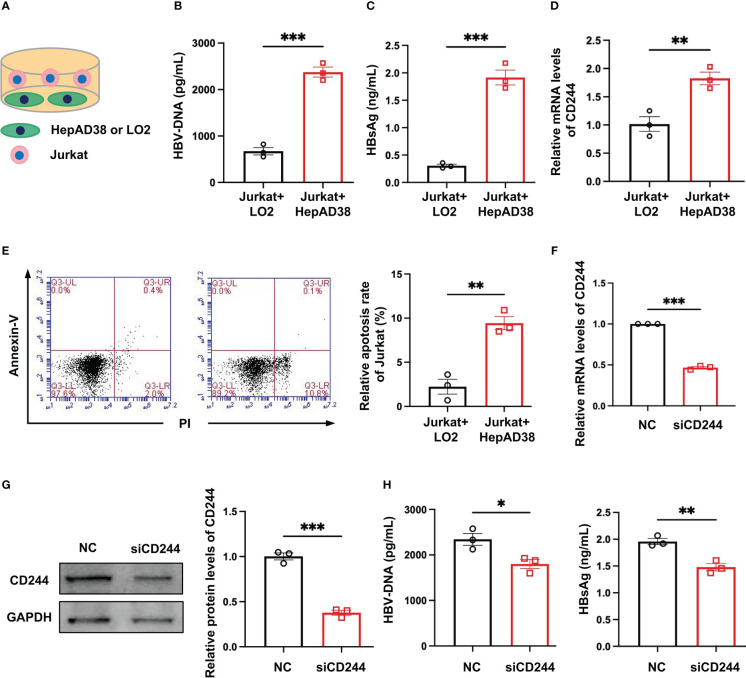
CD244 expression regulated T cells apoptosis with or without infection of HBV. **(A)** The co-culture scheme of LO2 hepatocytes or HBV infected HepAD38 cells with Jurkat cells. **(B)** HBV DNA detection in co-culture cells using ELISA. **(C)** HBsAg detection in co-culture cells using ELISA. **(D)** RT-PCR analysis of CD244 expression in Jurkat cells. **(E)** The apoptosis of Jurkat cells were identified by Annexin V/PI staining. **(F, G)** The expression of CD244 mRNA levels **(F)** and protein levels **(G)** in Jurkat cells after siCD244 transfection of co-cultured system. **(H)** Quantification of HBV DNA and HBsAg by ELISA kit. Data (n = 3 per group) were expressed as mean ± SEM and analyzed using Student t test, * *P* < 0.05, ** *P* < 0.01, *** *P* < 0.001.

In order to define the roles of regulating the differential expression of CD244 in T cells of CHB and SC HBV, we established an *in vitro* model. We co-cultured Jurkat cells, a T cell line, with LO2, a normal hepatocyte line, or HepAD38 cells, a hepatocyte line infected with HBV (**Figure 3A**). Then, HBV DNA and HBsAg were assayed to prove the HBV infection in HepAD38 cells co-cultured with Jurkat cells **(** **Figures 3B, C** **)**. CD244 was increased in Jurkat cells co-cultured with HepAD38 cells than co-cultured with LO2 cells (**Figure 3D**). The increasing of CD244 was associated with T cells apoptosis in Jurkat cells co-cultured with HepAD38 cells (**Figure 3E**), accompanied by higher HBV DNA and HBsAg. On the contrary, the levels of HBV DNA and HBsAg were significantly decreased after the silence of CD244 (**Figures 3F–H**). These data collectively suggested the importance of CD244 signaling in regulating CD8^+^ T cell immune responses during HBV infection.

### 3.4 The miR-330-3p enhanced immune response to HBV infection *via* inhibiting CD244 expression

**Figure 4:**
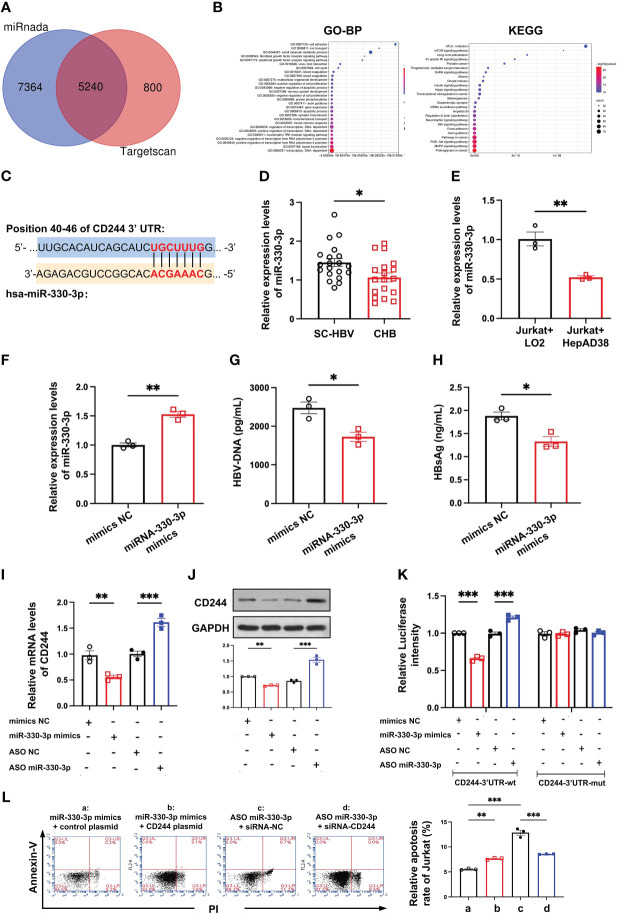
The miR-330-3p regulated immune response to HBV infection with CD244 alteration. **(A)** The target mRNAs predicted using the miRnada (www.microrna.org) and TargetScan (www.targetscan.org). **(B)** GO (biological process) and Kyoto Encyclopedia of Genes and Genomes (KEGG) pathway enrichment analysis. **(C)** The prediction interaction sites of CD244 and miR-330-3p. **(D)** RT-PCR analysis of miR-330-3p expression in CHB (n = 18) and SC HBV (n = 20) patients. **(E)** The expression of miR-330-3p in co-culture system of LO2 hepatocytes or HBV infected HepAD38 cells with Jurkat cells. **(F-H)** The alteration of miR-330-3p **(F)**, HBV DNA **(G)**, and HBsAg **(H)** treated with miR-330-3p mimics or negative control miRNA in the co-culture system of HepAD38 cells with Jurkat cells. **(I, J)** The expression of CD244 mRNA levels **(I)** and protein levels **(J)** in co-culture system after miR-330-3p mimics/ASO miR-330-3p (inhibitors) or controls transfection. **(K)** The relative luciferase activity in co-culture HepAD38 cells and Jurkat cells transfected with the indicated CD244-3’UTR-wt plasmid or indicated CD244-3’UTR-mut plasmid after the intervention of miRNA mimics/ASO miR-330-3p or not. **(L)** The rescue effect of siRNA-CD244 on the inhibition of apoptosis of Jurkat cells, inducing by CD244 plasmid or ASO miR-330-3p, by flow cytometry staining with Annexin V/PI. Data (n = 3 per group) were expressed as mean ± SEM and analyzed using Student t test or two-way analysis with Turkey’s multiple comparisons test, * *P* < 0.05, ** *P* < 0.01, *** *P* < 0.001.

To find the cause of regulating the differential expression of CD244 in SC HBV and CHB, we analyzed the DE miRNAs. In total, we predicted 5240 target mRNAs using the miRnada and TargetScan tools (**Figure 4A**). GO classification and KEGG pathway analysis of these DE miRNAs showed that transcription and immune pathway significantly increased (**Figure 4B**). Moreover, bioinformatics software TargetScan (http://www.targetscan.org) predicted that the target gene of DE miR-330-3p might be CD244 (**Figure 4C**). Then, we compared the level of miR-330-3p in CHB patients and SC HBV patients. RT-qPCR data showed that the expression of miR-330-3p was significantly decreased in CHB patients (**Figure 4D**). Furthermore, the co-cultured LO2 or HepAD38 cells with Jurkat cells showed that the expression of miR-330-3p was significantly decreased in HBV infection group (**Figure 4E**), accompanied by the higher level of CD244 (**Figure 3D**). On the contrary, HBV DNA and HBsAg were significantly decreased after stimulation of miR-330-3p mimics in HepAD38 cells co-cultured with Jurkat cells (**Figures 4F–H**).

To present the interactions between has-miR-330-3p and the mRNA of CD244, the co-cultured Jurkat cells and HepAD38 cells were transfected with mimics NC, has-miR-330-3p mimics, ASO NC, and has-miR-330- 3p ASO, respectively. After verifying that mimics and ASO of miR-330-3p work properly, we found that miR-330-3p mimics decreased the mRNA and protein level of CD244, while miR-330-3p ASO significantly increased the expression of CD244 (**Figures 4I, J**). In order to further explore whether miR-330-3p targeted CD244 *via* direct binding, a double luciferase test was designed. The results showed that decreased expression of CD244 induced by miR-330-3p mimics and increased expression of CD244 induced by miR-330-3p ASO in CD244-3’UTR-WT group were eliminated by CD244-3’UTR-Mut (**Figure 4K**). Furthermore, the apoptosis of CD8^+^ T cells was significantly increased in the overexpression of CD244 by CD244 plasmid transfection and the knockdown of miR-330-3p in ASO-miR-330-3p transfection (**Figure 4L**). Inhibition of CD244 by siRNA rescued the apoptosis of T cells, which were induced by ASO-miR-330-3p (**Figure 4L**). The results suggested that has-miR-330-3p targets CD244 *via* direct interaction.

### 3.5 Lnc-AIFM2-1 acted as a ceRNA for miR-330-3p to regulate CD244 expression

**Figure 5:**
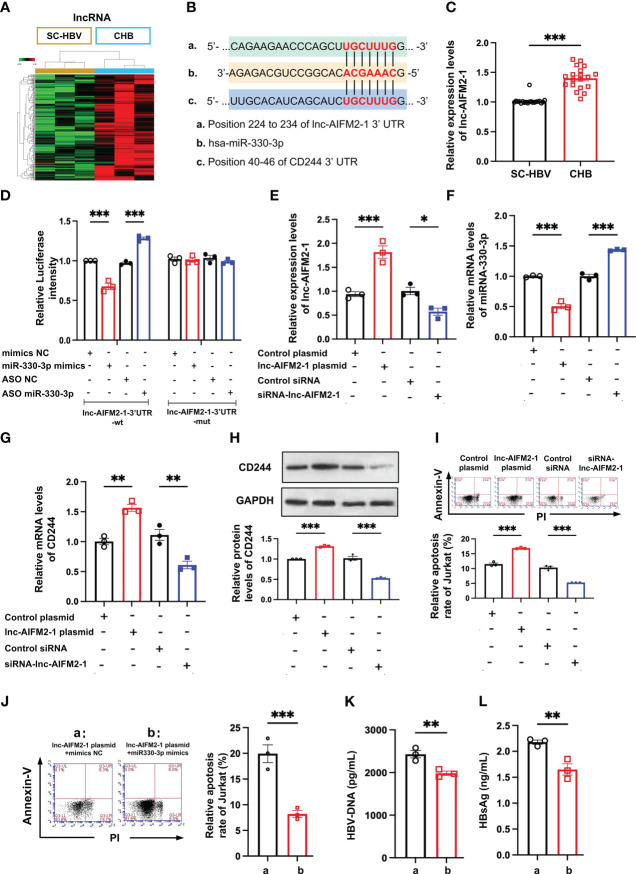
The interaction between lnc-AIFM2-1 and miR-330-3p regulated the immune response. **(A)** Heat map of up-regulated lncRNAs in CHB (n = 3) patients compared with SC HBV (n = 3) controls. Screening criteria were as follows: *P* ≤ 0.05 for lncRNAs. Expression values are depicted in line with the color scale; intensity increases from green to red. **(B)** The prediction interaction sites of CD244 with miR-330-3p and lnc-AIFM2-1. **(C)** RT-PCR analysis of lnc-AIFM2-1 expression in CHB (n = 18) and SC HBV (n = 20) patients. **(D)** The relative luciferase activity in co-culture HepAD38 cells and Jurkat cells transfected with the indicated lnc-AIFM2-1-3’UTR-wt plasmid or indicated lnc-AIFM2-1-3’UTR-mut plasmid after the intervention of miRNA mimics/ASO miR-330-3p or not. **(E, F)** The expression of lnc-AIFM2-1 **(E)** and miR-330-3p **(F)** in co-culture system of LO2 hepatocytes or HBV HepAD38 cells with Jurkat cells after lnc-AIFM2-1 plasmid/siRNA-lnc-AIFM2-1 or controls transfection. **(G, H)** The expression of CD244 mRNA levels **(G)** and protein levels **(H)** in co-culture system after lnc-AIFM2-1 plasmid/siRNA-lnc-AIFM2-1 or controls transfection. **(I)** The apoptosis of Jurkat cells were identified by Annexin V/PI staining in co-culture HepAD38 cells and Jurkat cells transfected with the indicated lnc-AIFM2-1 plasmid/siRNA-lnc-AIFM2-1 or not. **(J-L)** The rescue effect of miR-330-3p mimics on the inhibition of apoptosis of Jurkat cells, inducing by lnc-AIFM2-1 plasmid, by flow cytometry staining with Annexin V/PI **(J)**, and quantification of HBV DNA **(K)** and HBsAg **(L)** detected by ELISA. Data (n = 3 per group) were expressed as mean ± SEM and analyzed using Student t test or two-way analysis with Turkey’s multiple comparisons test, * *P* < 0.05, ** *P* < 0.01, *** *P* < 0.001.

LncRNAs are emerging as important regulators in the modulation of virus infection by targeting mRNA transcription (25). Integrating the lncRNA/miRNA interactions with the miRNA/mRNA interactions, the heat maps of these RNAs indicated that the upregulated lncRNAs can distinguish between CHB patients and SC HBV patients (**Figure 5A**). We found that lnc-AIFM2-1 and CD244 exited similar binding sites with miR-330-3p (**Figure 5B**). Therefore, we compared the level of lnc-AIFM2-1 in CHB patients and SC HBV patients. RT-qPCR data showed that the expression of lnc-AIFM2-1 was significantly increased in CHB patients (**Figure 5C**). To present the directly interactions between has-miR-330-3p and lnc-AIFM2-1, a double luciferase test was designed. The results showed that miR-330-3p mimics significantly suppressed the expression of lnc-AIFM2-1 and miR-330-3p ASO increased the expression of lnc-AIFM2-1, which were inhibited by lnc-AIFM2-1-3’UTR-Mut (**Figure 5D**). Moreover, we overexpressed the lnc-AIFM2-1 by transfecting the lnc-AIFM2-1 plasmid and deleted the expression of the lnc-AIFM2-1 by transfecting the lnc-AIFM2-1 siRNA (**Figure 5E**). We found that upregulation of lnc-AIFM2-1 induced the decreased miR-330-3p and the increased CD244 (**Figures 5F, G**). The protein expression changes of CD244 were consistent with mRNA (**Figure 5H**). In addition, the downregulation of lnc-AIFM2-1 induced the increased miR-330-3p and decreased CD244 (**Figures 5F, G**). Accompanying by the elevated CD244, the apoptosis of CD8^+^ T cells was significantly increased in the overexpression of lnc-AIFM2-1 group (**Figure 5I**). On the contrary, the apoptosis of CD8^+^ T cells was decreased in the lnc-AIFM2-1 siRNA group, accompanying by the decreased CD244 (**Figure 5I**). Furthermore, the apoptosis of CD8^+^ T cells were significantly inhibited by the treatment of miR-330-3p mimics, comparing with the group of lnc-AIFM2-1 plasmid+mimics NC (**Figure 5J**). The levels of HBV DNA and HBsAg were also significantly decreased in the treatment of miR-330-3p mimics than the group of lnc-AIFM2-1 plasmid+mimics NC (**Figures 5K, L**). These data suggested that lnc-AIFM2-1 acted as ceRNA for miR-330-3p to contribute to HBV immune escape.

## 4 Discussion

HBV infection and CHB caused by HBV is global public health problems (26). CHB patients are at a significantly increased risk of developing liver failure, cirrhosis, and HCC (27). However, the mechanisms by which HBV evades host immunity and sustains chronic infection are not fully understood. CD8^+^ T cells directly suppress viral replication and subsequent host dissemination by eliminating infected cells (28). In addition to TCR-mediated Ag recognition and pathogen clearance, CD244 is expressed on T cells and interact with their ligands on antigen-presenting cells upon TCR ligation, resulting in modulation of the T cell response (29, 30). CD244 is upregulated on CD8^+^ T cells during HBV infection, and CD244 signaling reduces production of IFN-γ by CD8^+^ T cells (8). The role of CD8^+^ T cells in anti-HBV immunity led us to examine the expression of molecules associated with the CD244 signaling pathway in CD8^+^ T cells during active HBV infection. In this study, samples from patients with CHB and patients with SC HBV were analyzed by flow cytometry. The results showed that the expression of CD244 on CD8^+^ T cells in CHB was significantly increased, which was consistent with the previous report, indicating that the abnormally high expression of CD244 was related to the chronicity of HBV.

Recently, miRNAs function in RNA silencing and post-transcriptional regulation of gene expression, and have received much attention in HBV infection (16). Previous studies demonstrated that chronic inflammation and/or viral factors can induce increased expression of miR-146a, which depresses T-cell immune function by targeting STAT1, in T cells in CHB patients (31). Here, we used microarray analysis of *miRNA* expression in CHB and SC HBV patients and further used two bioinformatics databases (miRDB and TargetScan) to speculate potential target genes for miR-330-3p, and found CD244 may be a target gene of miR-330-3p. Previous studies showed that *miR*-*330*-*3p* played an important role in the development of multiple tumors (32, 33). Moreover, miR-330-3p down-regulates the RNA level of mitogen activated protein kinase 1 (MAPK1) in liver cancer cells, thereby inhibiting the migration of liver cancer cells (34). We found that miR-330-3p was decreased in CHB patients and further determined the direct interaction between miR-330-3p and CD244, which increased CD8^+^ T cell apoptosis and HBV immune escape. Perhaps miR330 is one of the promoting factors for chronic hepatitis B patients to develop into liver cancer, which needs further research.

Recent studies have shown that the lncRNA can extensively participate in many biological processes such as cell signal transduction and immune response through the mechanisms of epigenetic modification, transcriptional and post transcriptional regulation (35). The abnormal function of lncRNA is closely related to the occurrence and development of many diseases. The study reported that lncRNA-HULC is highly expressed in patients with CHB and hepatitis B related liver cancer, and can promote the proliferation of liver cancer cells by downregulating the tumor suppressor gene *p18 (* 36, 37). In addition, Feng et al. reported that lncRNA PCNAP1, as the sponge of miR-154, regulates the proliferating cell nuclear antigen (PCNA), thereby promoting HBV replication and hepatocarcinogenesis (17). In this study, we expected to screen out the lncRNAs related to miR-330-3p, and use microarray analysis to screen out the differentially expressed lncRNAs in CHB and SC HBV patients. The bioinformatics method was used to predict the interaction targets of lnc-AIFM2-1 and miR-330-3p. Lnc-AIFM2-1 is an antisense chain located on chromosome chr10:69994626-70007836 (hg38), belonging to the intergenic lncRNA, containing three exons and a total length of 524 nt. ORF Finder and Reg RNA 2.0 software predict that it has no open reading frame and no ability to encode protein.

There are various interactions between miRNA and lncRNA to participate in the occurrence and development of diseases. As previous studies have reported that with the miRNA response elements, lncRNA could compete with mRNA to bind with miRNA, thereby freeing mRNA from the regulation of miRNA (38). There has been substantial interest in the ceRNA hypothesis in recent years, with much of the research in the area revolving around how dysregulation of ceRNA expression can affect diseases pathogenicity and progression (39). Recently, the study offers evidence that the lncRNA TUG1-miR-328-3p-SRSF9 mRNA axis function as a novel ceRNA regulatory axis, which may be associated with HCC malignancy and may be one of therapeutic targets of the anti-HCC treatment (40). According to the ceRNA hypothesis, to determine whether the lnc-AIFM2-1 acts as a ceRNA for miR-330-3p, we first examined the alterations of lnc-AIFM2-1 in CHB patients that occur CD244 upregulated and miR-330-3p downregulated. Our results demonstrated that miR-330-3p could suppress the luciferase activity of lnc-AIFM2-1, indicating the interaction between lnc-AIFM2-1 and miR-330-3p. Then, we further found that the decreasing of HBV clearance and increase of CD8^+^ T cells apoptosis with CD244 upregulated during lnc-AIFM2-1 overexpression. Finally, miR-330-3p transfection can rescue the CD8^+^ T cells apoptosis caused by overexpression of lnc-AIFM2-1. The results showed that lnc-AIFM2-1 and miR-330-3p play a critical role in CHB.

In summary, this study demonstrates that lnc-AIFM2-1 on CD244 by acting as a ceRNA of miR-330-3p contributes to HBV immune escape. This effect is due to the competition between lnc-AIFM2-1 and miR-330-3p to inhibit the expression of CD244 on CD8^+^ T cells, which are key immune responses to HBV. These data provide novel insights into the roles of interaction networks among lncRNA, miRNA, and mRNA in HBV immune escape. Furthermore, these findings suggest that lnc-AIFM2-1 and CD244 may be novel targets for diagnosis and treatment in CHB.

## Data availability statement

The datasets presented in this study can be found in Gene Expression Omnibus (GEO) with accession number GSE224283 (https://www.ncbi.nlm.nih.gov/geo/query/acc.cgi?acc=GSE224283).

## Ethics statement

The studies involving human participants were reviewed and approved by Research Ethics Committee of West China Hospital of Sichuan University. The patients/participants provided their written informed consent to participate in this study.

## Author contributions

CX: Conceptualization, Formal analysis, Data curation, Writing - original draft. SW: Conceptualization, Methodology, Investigation, Formal analysis. HZ, YZ, and PJ: Data curation, Validation. SS and YS: Validation. JC: Supervision, Project administration, Writing - review and editing. All authors contributed to the article and approved the submitted version.
