# Supplementary material for: Lnc-AIFM2-1 promotes HBV immune escape by acting as a ceRNA for miR-330-3p to regulate CD244 expression
Source: Front Immunol. 2023 Feb 9;14:1121795. doi: 10.3389/fimmu.2023.1121795 (PMC9946971; doi:10.3389/fimmu.2023.1121795)
Supplement: Supplementary file 1 [file DataSheet_1.docx]

## Supplementary information


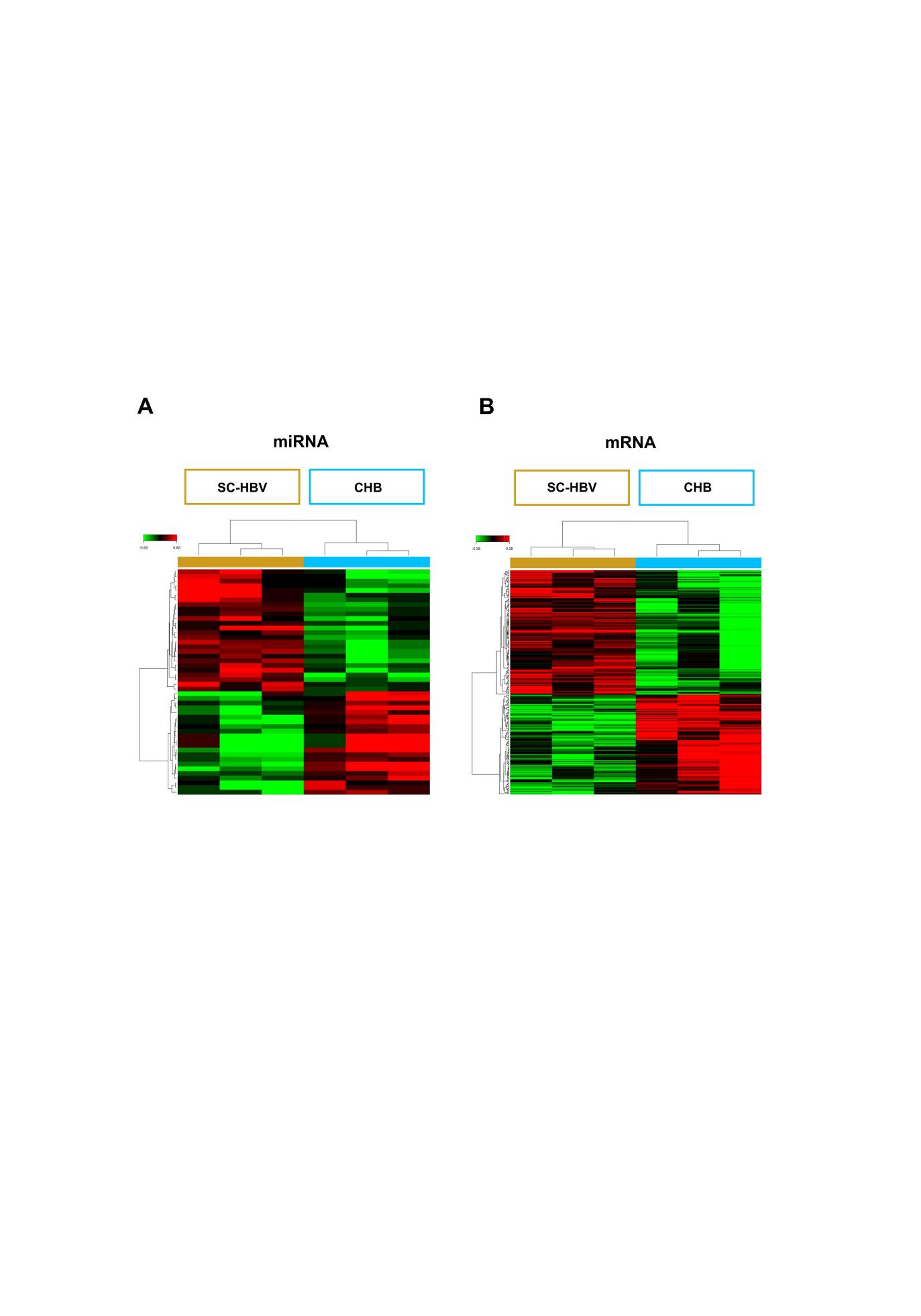


**Supplementary Figure 1. Heat map of differentially expressed miRNAs and mRNAs between CHB patients and SC HBV controls.** Screening criteria were as follows: *P ≤* 0.05 for miRNAs (A) and mRNAs (B). Expression values are depicted in line with the color scale; intensity increases from green to red. Each column represents one sample of CHB (n = 3) and SC HBV (n = 3) patients, and each row indicates a transcript.


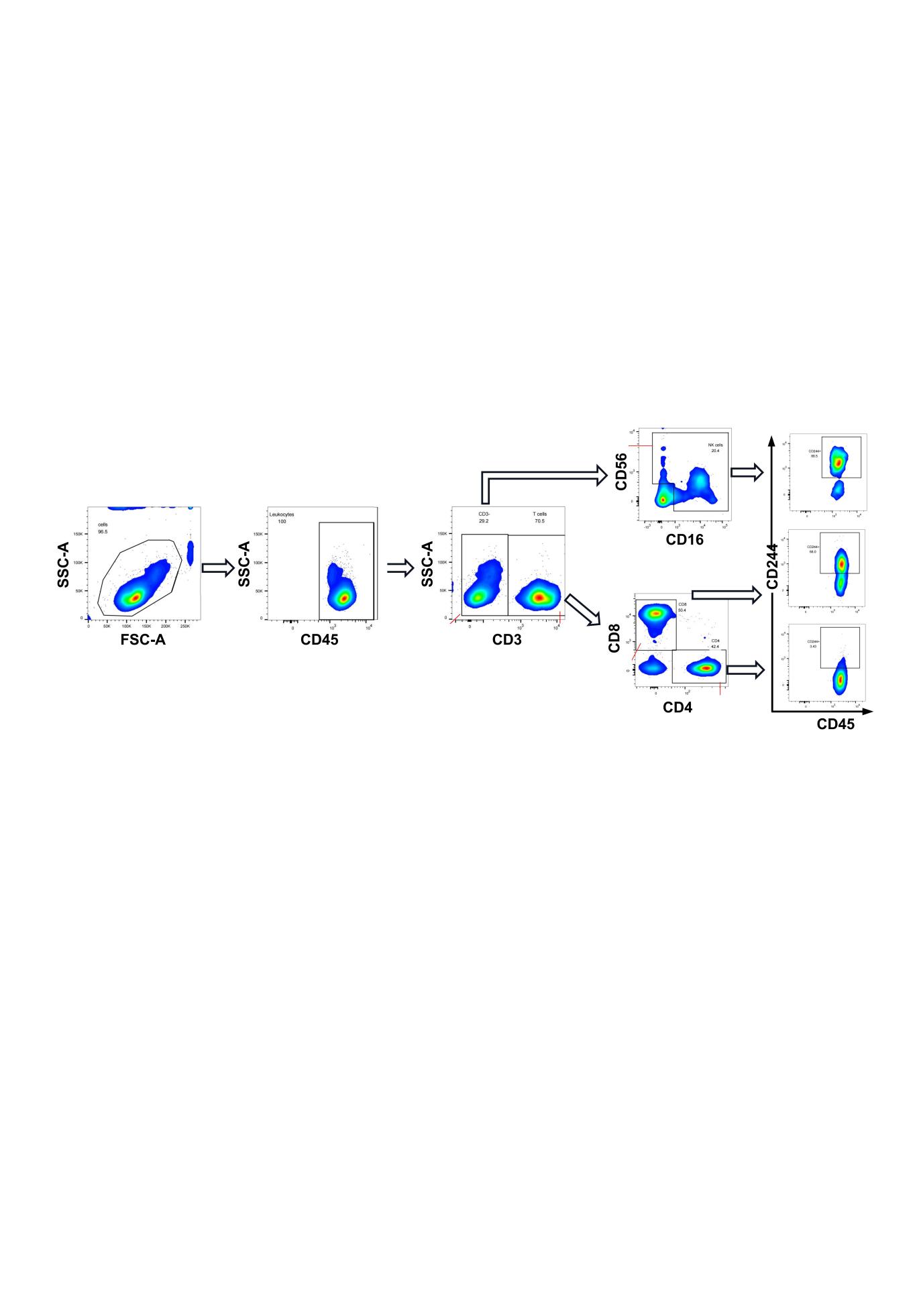


**Supplementary Figure 2. Representative flow cytometry plots showing gating strategy for CD244+ cells in the PBMCs.** FSC, forward scatter; SSC, side scatter.

**Supplementary Table 1**

**Clinical characteristics of patients**

| **Index** | **SC-HBV** | **CHB** |
| --- | --- | --- |
| Age （year） | 50 ± 6 | 43 ± 8 |
| HBsAg (IU/mL) | 0.40 ± 0.05 | 1361.96 ± 3527.49 |
| HBeAg (NCU/mL) | 0.2 ± 0.2 | 48.7 ± 216.5 |
| HBeAb NCU/mL) | 1.204 ± 0.291 | 0.495 ± 1.034 |
| HBcAb NCU/mL) | 0.101 ± 0.204 | 0.007 ± 0.002 |
| TBIL (μmol/L) | 12.3 ± 4.3 | 14.0 ± 5.3 |
| DBIL (μmol/L) | 3.2 ± 1.1 | 4.0 ± 1.6 |
| GLU (mmol/L) | 4.95 ± 0.63 | 4.94 ± 0.53 |
| TBA (μmol/L) | 2.7 ± 1.1 | 4.7 ± 6.9 |
| AST (IU/L) | 36.3 ± 38.3 | 26.6 ± 13.2 |
| ALT (IU/L) | 26.7 ± 14.9 | 24.1 ± 5.2 |
| ALP (IU/L) | 72.5 ± 22.1 | 80.7 ± 19.8 |
| GGT (IU/L) | 44.0 ± 39.6 | 19.7 ± 8.4 |
| TP (g/L) | 72.0 ± 3.5 | 74.2 ± 4.8 |
| ALB (g/L) | 47.1 ± 3.8 | 47.6 ± 4.0 |
| GLB (g/L) | 25.3 ± 3.0 | 26.5 ± 3.7 |
| TG (mmol/L) | 1.96 ± 1.37 | 1.39 ± 0.85 |

**Supplementary Table 2**

**The qRT-PCR primer sequences**

| Primer Name | Forward sequence (5’ to 3’) | Reverse sequence (5’ to 3’) |
| --- | --- | --- |
| CD244 | CTCACCTACCTGGACGAGGA | CCAAAAACGGCCAAAATCT |
| hsa-miR-330-3p | GCGAGCGCAAAGCACACGGCCU | GCAGGGTCCGAGGTATTC |
| U6 | CTCGCTTCGGCAGCACA | AACGCTTCACGAATTTGCGT |
| siRNA NC | UUCUCCGAACGUGUCACGUTT | ACGUGACACGUUCGGAGAATT |
| siRNA1 | UGGAUUUGUACCAUUCUUCUG | GAAGAAUGGUACAAAUCCAAG |
| siRNA2 | ACUCAUUGGUUCCUUUAAGGG | CUUAAAGGAACCAAUGAGUCC |
| siRNA3 | UCAUAUUCUGAAUCUCAUCCU | GAUGAGAUUCAGAAUAUGAAG |
| lnc-AIFM2-1 | CAGCATACACGGACTCACACA | GGCAACATCGCTCAAACTTA |
| GAPDH | CGAGATCCCTCCAAAATCAA | TTCACACCCATGACGAACAT |
